# Impact of Unsolicited Negative Feedback in Academic Settings

**DOI:** 10.7759/cureus.43640

**Published:** 2023-08-17

**Authors:** Shazia Sheikh, Fauzia Nausheen, Joel Arvizo-Zavala, Sherif S Hassan

**Affiliations:** 1 Education, California University of Science and Medicine, Colton, USA; 2 Medical Education, California University of Science and Medicine, Colton, USA; 3 Anatomy, Faculty of Medicine, Cairo University, Cairo, EGY; 4 Medical Education, Anatomy, and Neuroanatomy, California University of Science and Medicine, Colton, USA

**Keywords:** unsolicited comments, self-criticism, performance, mental well-being, negative feedback

## Abstract

Introduction: Appropriate feedback is crucial for quality improvement, productivity, and growth. There is a scarcity of information on the effect of unsolicited negative feedback (USNF). Our current study aimed to investigate the impact of USNF on motivation, performance, and mental well-being.

Methods: Data was collected on a 5-point Likert scale by a survey of 10 validated questions asking the impact of USNF around three themes: 1. “Mental Health,” 2. “Motivation,” and 3. “Performance.” The rationale of the questions was to investigate the negative emotional state and its impact on confidence, motivation, and performance of similar activities after getting USNF. Additionally, it impacts the mental state of anger, sadness, and self-criticism.

Results: A total of 38 participants completed the questionnaire. The agreement after USNF was 57.8% to develop harsh or critical self-talk, 76.3% for mood changes, 57.8% helps to learn, and 63% helps to fix things received on positive feedback. A total of 86.8% need time to reflect, 76.3% need time to process, 65.78% try to avoid USNF, 31.57% start to avoid people who give USNF, and 23.68% try to prove themselves to those who give USNF. The results showed a strong correlation between a person's need for time to process emotions and demand for space to process emotions and forecasting memories after the USNF.

Conclusion: The psychological implications of USNF can be profound, leading to long-term negative effects on mental health, motivation, and performance. Training individuals to deliver negative feedback in a more constructive and positive manner is essential to mitigate these detrimental consequences.

## Introduction

Feedback plays a crucial role in evaluating faculty by the medical students and medical students by the faculty, serving as a vital component of performance training. While the act of providing feedback is recognized as essential, it is equally imperative for faculty and students to receive and internalize feedback actively. Embracing feedback positively facilitates their growth, development, and the potential for meaningful behavior change, ultimately enhancing their skills and competencies in the academic and medical domains [1,2]. Numerous research has been conducted to identify the various methods of offering feedback. However, much work hasn’t been put into determining the impact of unsolicited negative feedback (USNF) on self-rated assessment, affective reactions, learning motivation, performance, and mental health, particularly in academic medicine [3].

Feedback is given to enhance the learner's performance and is seen as the transfer of knowledge gathered through direct observation [1]. In most academic contexts, receiving feedback is required for the training and education of medical students and resident physicians and faculty and staff evaluation. According to some theories, providing and receiving feedback is the key to effective workflow and, ultimately, high-quality patient care [2,4]. In typical academic environments, there are two kinds of feedback: The first kind is informal feedback, which is composed of numerous talks between numerous individuals within an organization. This kind of feedback is especially concentrated on objectives, performance, behaviors, and actions to accomplish these objectives. The second feedback is formal feedback, which entails formal yearly assessments between a faculty member or professor and the students they supervise [2,4]. Feedback can be given in various ways, one of which is expressing gratitude for someone's work and praising them for it. Coaching and advising someone to advance their knowledge and abilities is another form. Evaluation, the final form, gauges an individual's performance. One of the fundamental components of mentoring, coaching, supervising, and parenting is providing feedback on performance. It is regarded as one of the most effective educational exercises and helps to resolve disagreements in close relationships. Feedback can be categorized as positive or negative. Positive feedback is a reaction to achieving specific desired outcomes from specific tasks. This kind of feedback aims to strengthen that response and support further efforts taken to accomplish a certain objective while motivating that person to perform better. Positive and corrective feedback is the ideal method of completing novel activities, according to Simonian and Brand [5-7].

Negative feedback is a reaction to tasks producing bad results when adjustments are needed. According to Oktaria and Soemantri's research, [8] positive feedback improves performance more than negative feedback, especially when it comes from an unexpected source. The most frequent reason for instructors and students in the medical field to avoid receiving feedback is because of worries and worry that can be caused by the evaluators' critical remarks [8]. The accuracy of self-assessment, as well as feelings and self-efficacy, is significantly influenced by verbal input from evaluators. When giving feedback to students, instructors should be mindful of the potential effects of both positive and negative comments [9]. Positive and constructive feedback can develop self-confidence oneself, whereas USNF can have a damaging effect. In comparison, constructive feedback that is delivered as positive criticism has been proven to be more effective at improving skills than just positive compliments. A delicate distinction exists between positive criticism and detrimental critical feedback, as individuals tend to react adversely to the latter. The performance can worsen after receiving critical feedback. According to the National Institutes of Health (NIH) [10,11] and other studies, women and people of color are more frequently and disproportionately subjected to critical remarks, which has a negative impact on workplace diversity. Although the recipient of any kind of feedback is likely to experience strong emotional reactions, unwelcome USNF can have a detrimental effect that can harm mental health. It is crucial to be aware of and sensitive to the emotional reactions of the recipients because they can impair performance and encourage unproductive conduct.

In the current study, we hypothesized that positive and constructive feedback could positively impact performance, motivation, and mental well-being, while USNF can cause anxiety and low performance due to a lack of confidence and reduced motivation due to fearfulness. The objective is to study the impact of USNF on faculty and staff at a medical school and its impact on their motivation, performance, and mental well-being.

## Materials and methods

The institutional review board (IRB) approved the research, and IRB exemption was approved: #: HS-2022-13. We conducted a survey by asking the faculty and staff to complete a questionnaire about their opinion around three themes: The impact of USNF on the following: 1. “Mental Health,” 2. “Motivation,” and 3. “Performance.” Their responses were collected using a 5-point Likert scale. This scale consisted of five options: 5 = "Strongly Agree," abbreviated as "S AG," 4 = "Agree," abbreviated as "AG," 3 = "Neither Agree nor Disagree," abbreviated as "N AG nor DAG," 2 = "Disagree" abbreviated as "DA," and 1 = "Strongly Disagree" abbreviated as "S DA." This scale helped them express their level of agreement or disagreement with the statements in the study.

The first question (Q.1) asked about their consent to participate in the study, and after their agreement, the validated survey questions started.

Q.2. After receiving USNF from a peer or colleague, I am prone to harsh or critical self-talk. The rationale of this question was to investigate the mental state of getting negative emotions and its impact on confidence and performance. Similarly, all other questions below were asked to achieve a certain objective.

Q.3. I am prone to harsh or critical self-talk after receiving USNF from a peer or colleague; my mood may change as a result. The rationale for this question was to explore the impact of USNF on an individual's self-talk and mood to get an insight into the psychological repercussions of critical remarks in a workplace setting.

Q.4. I am prone to remember the other instances of negative feedback when I receive new USNF. The rationale for this question is to investigate how recollecting past negative feedback may influence an individual's response and perception of new USNF.

Q.5. I am prone to see negative feedback as a learning tool. The rationale for this question is to explore the individual's perspective on using negative feedback as a constructive learning tool rather than perceiving it as a negative experience.

Q.6. I tend to want to implement or fix things based on the positive feedback I receive. The rationale for this question is to understand the individual's inclination to take action and make improvements based on the positive feedback received.

Q.7. I may need time to reflect on the purpose of USNF. The rationale for this question is to gauge the individual's need for reflection and understanding of the purpose behind USNF.

Q.8. After receiving USNF, I may need time to process my feelings of sadness or anger. The rationale for this question is to assess the individual's emotional response and need for processing after receiving USNF.

Q.9. I try to avoid receiving USNF by ensuring I do things with extra care and precision. This question aims to understand the individual's approach to avoiding USNF by being cautious and meticulous in their actions.

Q.10. After receiving USNF, I may avoid the person who provided the feedback to me. The rationale for this question is to explore how individuals may react to USNF by potentially avoiding or distancing themselves from the person who gave the feedback.

Q.11. After receiving USNF, I may try to prove the person wrong by any means necessary. This question aims to understand how individuals may respond to USNF by feeling motivated to prove the feedback giver wrong through various actions and behaviors.

Q.12. Do you have any specific comments regarding the Impact of USNF on Mental Health, Motivation, and Performance? The rationale for this question is to gather specific insights and comments from individuals about how USNF may affect their mental health, motivation, and performance. This information can help in understanding the potential consequences of USNF and how it may impact individuals in the workplace or other settings.

At the end of the survey, a free-response question was posed. The free-response survey aimed to gather suggestions on how to make the feedback process for the three topics outlined above better moving forward.

## Results

The study's findings were evaluated based on the percentages of faculty members and staff who felt that unsolicited negative comments about their work had a detrimental impact on their motivation, performance, and mental health. Thirty-eight faculty and staff members consented, answered the questions, and completed the questionnaire. Following USNF, participants agreed that 57.8% of them acquired harsh or critical self-talk, 76.3% experienced emotional swings, 57.8% learned new things, and 63% used positive feedback to help them fix problems. A total of 86.8% required some reflection time, 76.3% required some processing time, 65.78% attempted to avoid giving USNF, 31.57% began to avoid those who gave USNF, and 23.68% attempted to win over those who gave USNF (Table 1).

**Table 1:** Percentage of responses to the questions SAG: Strongly Agree, AG: Agree, SDAG: Strongly Disagree, DAG: Disagree

Question number	Number of AG and SAG	Percentage of AG and SAG	Number of DAG and SDAG	Percentage of DAG and SDAG
Q2 Harsh Talk	22	57.8%	7	18.4%
Q3 Mood Changes	29	76.3%	7	18.4%
Q4 Memories PUF	22	57.8%	13	7.89%
Q5 Feedback Learning Tool	24	63%	10	26.31%
Q6 Implement FB Received	24	63%	4	10.52%
Q7 Time Reflection	33	86.8%	2	5%
Q8 Time Process Emotions	29	76.3%	3	7.89%
Q9 Avoid Feedback Precision	25	65.78%	6	15.78%
Q10 Feedback Avoidance	12	31.57%	18	47.36%
Q11 Feedback Prove wrong	9	23.68%	17	44.73%

Our findings suggest that the strongest positive correlation (r = 0.766) exists between changes in mood subsequent to receiving USNF and harsh self-talk after receiving USNF. These findings align with the idea that USNF can result in negative emotional or mental health consequences for participants, and this analysis supports that idea. Our findings also suggest multiple moderate positive correlations between two variables and multiple other variables - mood changes and time to process emotions.

Mood changes had positive correlations with harsh self-talk (r = 0.766), time for reflection (r = 0.545), and time to process emotions (r = 0.571). When considering the realities of harsh self-talk, the time needed to reflect, and the time to process emotions, it’s important to consider the mental health and emotional well-being of those who have received UNF. With two moderate correlators and one strong correlator with mood changes, it can be surmised that UNF affects mood and may also affect the internal processes one utilizes to cope with unsolicited advice.

**Figure 1:**
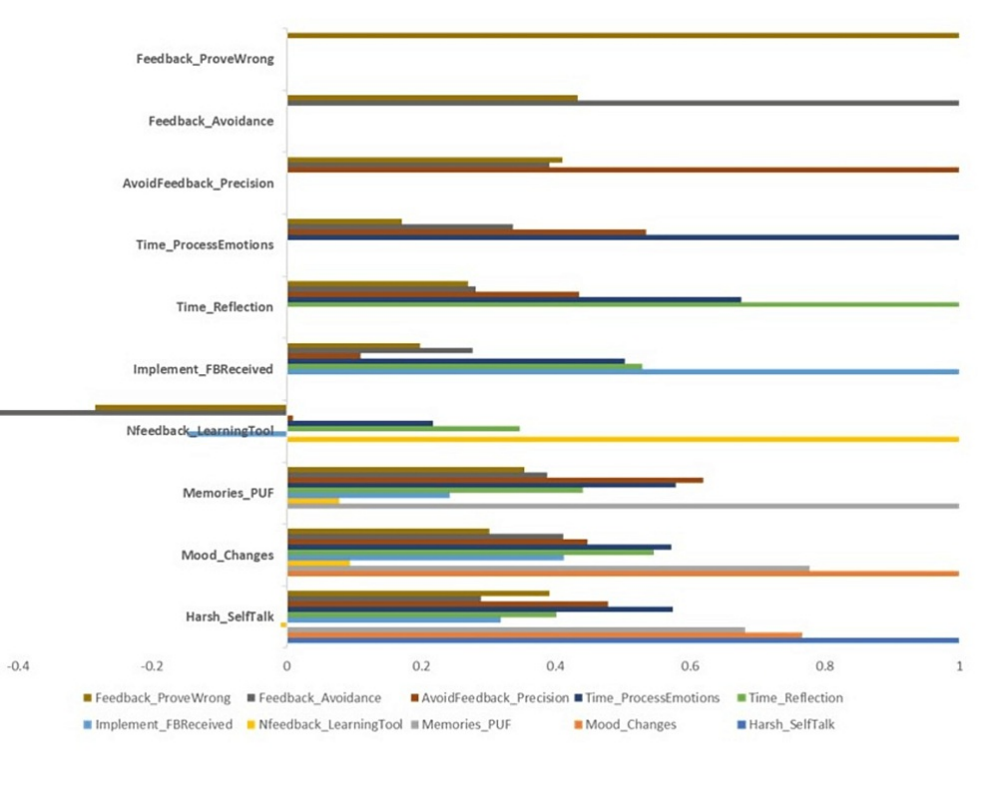
Correlation between the feedback and emotional state

Time to process emotions has positive correlations with harsh self-talk (r = 0.574), mood changes (r = 0.571), memories of previous USNF (r = 0.578), and a need for reflection time (r = 0.675). With four moderate correlators, it can be surmised that when one utilizes time to process emotions associated with UNF, internal emotional responses are affected in addition to potential changes in mood. What is also interesting about the positive correlations associated with time to process emotions is that two somewhat conflicting correlations arise. Time to process emotions was positively correlated with implementing feedback received (r = 0.502) and avoidance of UNF through perfectionist thinking or behaviors (r = 0.534). These results could potentially lead to further investigation and inquiry around whether the implementation of feedback received occurs through more immediate changes to avoid feedback in the future or if the implementation of feedback is a result of thoughtful reflection, given the positive correlation with the time needed to process emotions. Further inquiry is needed to understand better the internal processes - such as decision-making - used to determine how feedback is implemented after emotional processing (Figure 1).

Another interesting link exists between a person's need for space to process their emotions and forecasting memories of previously received uninvited remarks has a moderate correlation (r = 0.578). Although the correlation is moderate, researchers must be aware of how a person may engage in perfectionistic behavior to avoid USNF in the future. The potential behavioral ramifications of this may result in striving to avoid mistakes altogether or by processing the negative input differently as a preventative measure for any unpredictable discomfort associated with USNF.

An additional interpretation may be that people who take time to reflect on UNF may be trying to prove people wrong in the future regarding said feedback by implementing the feedback in a way to have less uncertainty and more predictability in not receiving UNF in the future. The distinction between ruminating on negative feedback and integrating it can be considered, and further inquiry is needed to understand better the internal thought processes and behavioral mechanisms that manifest, given these correlational results. The free responses of participants are shown in Table 2.

**Table 2:** Free responses regarding unsolicited negative feedback on three themes.

Themes	Open-Ended Response
Performance	Unsolicited Negative Feedback is unavoidable and reveals something about that individual's motives. If the motive is right then the feedback is far more valuable.
	Final thoughts... Unsolicited Negative Feedback can result in a set back in ones daily productivity or work agenda due to the time it takes to regroup, rethink, and proceed in a mannerly fashion to avoid hearing negativity.
Mental-well-being	My mental health, motivation, and performance diminish rapidly after receiving high volumes of Unsolicited Negative Feedback. On the other hand, fair criticism and objective negative feedback are easier to navigate as a way to improve oneself. I work very well with positive feedback, but I begin to believe in my own worthlessness and depreciated sense of self when showered with negative feedback, particularly from colleagues with supervisory roles.
	When I combat negative feedback I have learned to be a mirror and deflect. When receiving positive feedback I have learned to be a sponge.
	USNF tends to be detrimental for me as someone that also struggles with negative self-talk. It makes it challenging for me to want to continue to engage with that individual moving forward.
Motivation	It really depends on who is giving the feedback. Did I really think it was warranted, or was it just their impression or "feeling" that I did something to warrant negative feedback, so if I don't feel it is valid I really don't pay much attention to it.
	No
	It would be great to have a session that teaches employees how to respond to and manage unsolicited feedback.
	Negative feedback can be said in a more constructive way so as not to adversely effect health, motivation, and performance.
	It could dramatically effect your overall performance for a period of time.
	No one is perfect, however, I feel like unsolicited negative feedback makes me want to be. I tend to think about negative feedback for a while, to make sure it never happens again.
	No
	Unsolicited Negative Feedback mainly reflects a weakness in the person providing that negative feedback. It is not only lack of tact, but a reflection of the negative energy in the person speaking, not the one listening.
	The tone also matters. I appreciate a good tone when given Unsolicited Negative Feedback.
	If you could define “unsolicited negative feedback” that would help me answer. Additionally, even though I do have a righting reflex and initially dislike negative feedback, I believe it is important (when well-intentioned) for personal and professional development. I believe it is an important and necessary marker for deep, vulnerable, and mutually beneficial friendships. A lot of times, I can’t see what I’m doing or understanding wrong, but and helped to grow when my community kindly and gently points that out (even though because of the aforementioned lack of insight, I didn’t know to ask for it).
Non-specific	I think “negative feedback” is too broad of a term for me. I found myself answering a couple of survey questions with hesitation because I respond differently to supportive feedback for improvement vs. critical feedback. Does “negative feedback” include both of these types of feedback?
	If one is honest in one's endeavor, Unsolicited Negative Feedback is irrelevant.
	My reaction to Unsolicited Negative Feedback is highly dependent on the nature of the feedback. If the feedback is constructive and delivered privately, it is a great way to reflect and make positive changes. However, when feedback is not actionable and delivered publicly, I find it useless and emotionally straining.

Statistical methods

Correlation coefficients (r) were calculated for this study. Ten variables were under study, with three sets of variables resulting in positive correlations and two sets of variables resulting in negative correlations. The table below lists the variables and their respective positive and negative correlations (both moderate and strong). Those italicized are considered strong correlations. These numbers show how different factors are related to each other. These connections reveal how various factors are interrelated. For instance, when individuals experience shifts in their mood and engage in self-critical talk, there's a strong connection. Similarly, recollections of previous negative feedback combined with harsh self-talk also display a significant link. On a positive note, allowing time for emotional processing alongside practicing self-critical talk demonstrates a correlation. Conversely, certain negative associations exist, such as the connection between avoiding negative feedback through perfectionism and using negative feedback for learning, which has a notable link. These insights help unveil the complex web of relationships, showcasing both robust and subtle connections among diverse concepts (Table 3).

**Table 3:** Relationship of variables

Variable	Positive Correlation	Negative Correlation
Mood Changes and Harsh Self-Talk	0.766	
Memories of Previous Unsolicited Negative Feedback and Harsh Self-Talk	0.682	
Time to Process Emotions and Harsh Self-Talk	0.574	
Time for Reflection and Mood Changes	0.545	
Time to Process Emotions and Mood Changes	0.571	
Time to Process Emotions and Memories of Previous Unsolicited Negative Feedback	0.578	
Avoidance of Unsolicited Negative Feedback through Perfectionism and Memories of Previous Unsolicited Negative Feedback	0.619	
Time for Reflection and implementing feedback received	0.528	
Time to Process Emotions and implementing the feedback received	0.502	
Time to Process Emotions and time for reflection	0.675	
Avoidance of UNF through Perfectionism and time to process emotions	0.534	
Avoidance of Feedback and using Unsolicited Negative Feedback as a Learning Tool		-0.500

## Discussion

Feedback is defined as a series of remarks concerning a person's performance and the effort they are making to accomplish a task. A person's ability to complete a task and evaluate their own performance can both be improved with feedback [11]. Positive and negative feedback are the categories that are most frequently described. Positive feedback denotes the observer's declaration that the participant has attained the objectives and developed the desired abilities. Negative feedback, on the other hand, shows that improvement is still needed in order to change the skills or behavior to reach the necessary set of objectives [12]. The main goal of this research was to investigate the effects of negative feedback, particularly when it is unsolicited. The findings of our study are consistent with several studies that concentrate on providing and receiving feedback regarding someone's performance, abilities, or goal-achieving. Negative feedback, especially when it is unsolicited, might have a detrimental influence on performance in the future. This was the main emphasis of our study, which demonstrates that it has varying impacts [13]. The impact, however, differs depending on the feedback given, as evidenced by the fact that constructive criticism was discovered to be useful in improving certain talents that required corrective comments rather than merely positive compliments [14,15].

All forms of feedback might result in favorable or unfavorable emotional responses [16] that have an effect on our mental health. To prevent unproductive behaviors, it is crucial to consider the emotional reaction of the person receiving the feedback [17,18]. Our findings demonstrated that feedback should place more emphasis on what someone should improve upon (constructive feedback) and what they are doing right (positive feedback) rather than on what they are doing wrong (critical feedback). Additional guidance on what to do should be given to people rather than what not to do, highlighting their errors. The words "no" and "don't" should be eliminated. Being forthright is preferable [7]. Given the negatively correlated associations between USNF, learning tools, and feedback avoidance observed in this study, it's critical that participants receive training on how to express their negative feedback constructively. This training should replace USNF with a consent approach in which participants must request permission before sharing criticism with others. Training should emphasize that feedback should only be given when the recipient welcomes it and that it needs the participant to be an active agent in the discussion section [2].

The current study demonstrated that there were changes in mood following USNF and that receiving feedback was positively correlated with using harsh self-talk. This was a significant discovery because there was growing evidence linking harsh (or negative) self-talk to impulsive mood swings. Numerous writers, including Santos-Rosa et al. [19], who discovered that performance was negatively impacted by negative self-talk, support these findings. They observed that positive self-talk was a predictor of positive performance. The current study also demonstrated a positive correlation between a recipient's current experience with USNF and remembering earlier experiences with negative feedback, such as changes in mood and harsh self-talk. In other words, participants said that recalling those earlier experiences as a result of receiving USNF. One explanation for this is that recalling self-threatening negative input might have a motivating effect, which in turn serves the purpose of self-protection [20]. The results of the current study indicated that individuals who received USNF required time to think on their emotional reactions following an incident that might prolong those emotional reactions. These findings coincided with those of Morgan and Banerjee [21], who claimed that, at least for highly anxious individuals, a feeling of having failed in some way might lead to an overrepresentation of negative thoughts during rumination. A significant link existed between a person's demand for space to process their emotions and forecasting memories of previously received uninvited remarks. It can be predicted if someone will use perfectionism (or precision) to avoid negative feedback in the future if they have memories of receiving it in the past. This can be done by striving to avoid mistakes altogether or by processing the negative input differently.

People who need time to reflect on USNF predict trying to prove people wrong in the future regarding said feedback. In comparison, people who are not very expressive may take things very personally and may become offended. That feedback was unsolicited, so I will prove them wrong. The distinction between ruminating on negative feedback and integrating it can be considered.

The mood changes after the UNF were strongly related to self-harsh talk, and the revival of similar negative memories that impacted the mood negatively. After the negative impact on mood, time is needed to process and reflect on emotions after USNF because the impact on feelings was prolonged due to similar previous experiences. When people see USNF as a learning tool, this was negatively correlated with avoidance of future negative feedback, with less likely to avoid negative feedback in the future. When people see USNF as a learning tool, this is negatively correlated with trying to prove others wrong regarding given feedback, and they are more likely to act on the feedback in a non-retaliation sort of way. In the current study, the authors made the intriguing observation that people who recall past USNF can predict whether they will adopt perfectionism (or precision) to avoid negative feedback in the future. The likelihood of avoiding future negative feedback is adversely connected with using USNF as a learning tool, with a decreased likelihood of avoiding it. While attempting to disprove others' assertions about a given feedback is adversely connected with using USNF as a learning tool, acting on the criticism in a non-retaliatory manner is positively correlated. Providing creative examples of converting negative feedback into positive feedback could increase the efficacy of feedback as a learning tool [22].

Nonetheless, the types of personality can also have a huge impact on the approachability of negative comments and unsolicited feedback. Neuroticism personality people can have high sensitivity to negative comments and respond with high anxiety and self-doubt compared to people of agreeable traits who try to be considerate and compassionate. The limitation of this study is that we did not survey for the personality types and responses since most people are not aware of their type or do not want to disclose it [23]. The small sample size may limit the generalizability of the study which we will address in future studies.

## Conclusions

In conclusion, our study underscores the significance of feedback in improving performance and fostering self-improvement. It highlights the negative repercussions of USNF on motivation, mental well-being, and performance. The importance of providing constructive feedback as a valuable teaching tool is emphasized, focusing on improvement rather than criticism. Adopting a consent-based approach and providing training to deliver feedback constructively empowers recipients and reduces negative emotional responses. Understanding the relationship between mood changes and self-critical talk following negative feedback can aid in allowing individuals time to process their emotions. Furthermore, perceiving feedback as a learning tool is linked to more positive responses and better outcomes. Taking into account individual differences, particularly personality traits, can enhance the effectiveness of feedback delivery. In creating a culture of constructive feedback, we can foster a supportive and productive learning environment that benefits both feedback providers and recipients.
